# Anatomical and Morphological Structure of the Skull of a Juvenile Specimen of *Myotis myotis* (Chiroptera: Vespertilionidae)

**DOI:** 10.3390/ani14081225

**Published:** 2024-04-19

**Authors:** Grzegorz Kłys, Elżbieta Koenig

**Affiliations:** Institute of Biology, University of Opole, ul. Oleska 22, 45-052 Opole, Poland; elakoenig36@gmail.com

**Keywords:** cranium, greater mouse-eared bat, sutures, description of individual skull bones

## Abstract

**Simple Summary:**

There are few descriptions of bat skulls, and most are incomplete. In the representatives of the order Chiroptera, interosseous sutures disappear over a few months after birth. Conversely, the skull forms a nearly continuous structure in adult specimens, which makes analyzing it difficult. The work describes 28 bones, 30 bone sutures, 13 bone processes and 5 holes in the skull. The presence of the lacrimal bone, *os lacrimale*, was confirmed. The study concerned the structural elements of the skull and their sutures. The aim of this study was to produce a detailed description of the bat skull in order to fill in the gaps in knowledge on skeletal structures and provide descriptive material for use in anatomical research.

**Abstract:**

Few studies analyze the morphology and anatomy of the bat skull, and most of them are incomplete. Some of the difficulties stem from the fact that, in the representatives of the order Chiroptera, the interosseous sutures disappear by fusing together before active flight begins, which takes place over only a few months. This study presents a detailed morphological and anatomical description of the skull of a juvenile specimen of *Myotis myotis* (Borkhausen, 1797). Juvenile skulls are difficult to preserve and often incomplete. Previously inconsistent terminology related to bones, sutures, and other cranial structures was unified, which will provide insight on the distribution of each structure in both juvenile and adult specimens to be investigated. The description fill in the gaps in knowledge about the cranial structures of *Myotis myotis* and the representatives of the family Vespertilionidae. This will allow for precise descriptions of the skulls of bats.

## 1. Introduction

The Vespertilionidae (Chiroptera) are a cosmopolitan family populating the entire globe, apart from the Antarctic. Over 300 Vespertilionidae species have been described to date, including over 127 species from the genus *Myotis* [1]. Although bats constitute over 20% of known species of mammals, there are few detailed morphological and anatomical studies on the bat skull, and most of them are incomplete. Several publications contain extensive analyses of the cranial structure of both the suborder Megachiroptera [2,3,4] and the suborder Microchiroptera [5,6,7]; a number of older publications are also available [8,9,10,11,12,13,14,15,16], which are extremely valuable. In general, while these publications meet the current standards, they deviate from the expectations of modern science. It is difficult to describe species, both modern and extinct, without detailed skull anatomy. In recent years, researchers have focused on details such as teeth, the structure of the mandible, the general shape of the skull, selected elements of the skull, or statistical analyses of significant points in craniometric measurements [17,18,19,20,21]; meanwhile, studies on the skulls of young individuals, where individual bones are clearly visible, were omitted. Furthermore, other difficulties include inconsistent terminology related to bones, sutures and other cranial structures, and the fact that, in Chiroptera, interosseous sutures disappear over time, fusing before active flight begins [15]. Moreover, the skull does not fuse with the premaxilla and the nasal bone only in some representatives of the order; likewise, the tympanic bone does not fuse with the skull [17]. This makes it difficult to distinguish each bony element of the skull in adult specimens.

The aim of this study is to present a detailed morphological and anatomical description of the skull of a juvenile specimen of *Myotis myotis* (Borkhausen, 1797). Due to the vast amount of material, the authors decided to compare the distribution of unfused structures in the juvenile specimen with a fully ossified skull in a future publication.

The analysis will help to fill in the gaps in knowledge on the bony structures of *Myotis myotis* and related species.

## 2. Materials and Methods

The authors of this study used exhibits from the collection of the Upper Silesian Museum in Bytom (Poland) in order to present a detailed morphological and anatomical description of the skull of this species. Specifically, two juvenile and three adult specimens were used: exhibit numbers 6695/16425; 6695/16424; 6695/16426; 6695/16427; and 6695/16428. The remaining specimens served to complement the observed structures. The article contains macro photographs of the best-preserved skull. The specimens were obtained in Zagwiździe, Murów Municipality, Opole District, Opole Voivodship (N 50.874174, E 17.976379). The juvenile specimen selected for the study, aged 2–3 months, had both deciduous teeth and erupting permanent teeth. Macrophotographs were taken using a Canon EOS 750D digital camera. Six views of the skull of a selected individual were taken: dorsal, ventral, lateral, anterior, posterior and mandible. Each view was photographed in layers. Next, the layer file for each view was compressed into a single photograph using the Helicon Focus 8.2.2 software in order to obtain photographs with a high depth of focus. The background was edited to improve esthetics, and corrections were carried out in PhotoFiltre 7. Anatomical terminology was taken from Krysiak et al. [22] and Hermanson et al. [23]. For previously unnamed structures, new terminology was proposed that was not discussed in the reviewed publications. The description does not cover the hyoid apparatus (*apparatus hyoideus*). The description of the lateral wall of the skull uses the names squamosal (*os squamosum*) and temporal bone (*os temporale*) interchangeably.

## 3. Results

**Figure 1:**
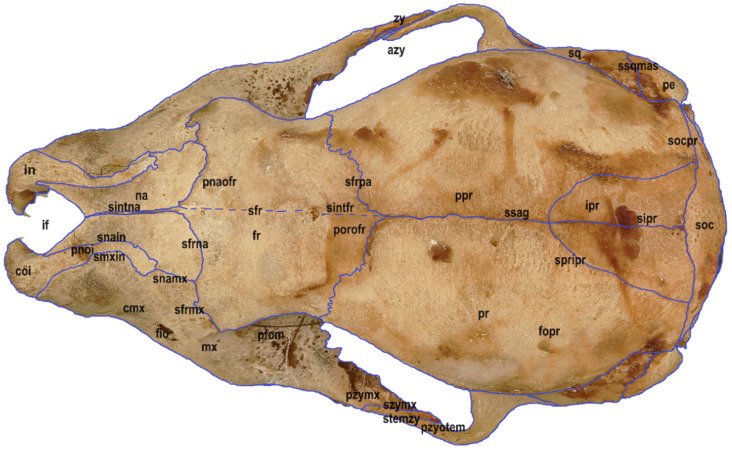
Dorsal view of the skull in a juvenile specimen of *Myotis myotis*, exhibit number 6695/1642. The bones, sutures, and fusions are marked with lines. Abbreviations are explained as follows: **in** *os incisivum*, **snain** sutura nasoincisiva, **smxin** sutura maxilloincisiva, **if** *incisive fissure*, **coi** *corpus ossis incisive*, **paoi** *processus alveolaris ossis incisive*, **pnoi** *processus nasalis ossis incisive*, **ppoi** *processus palatinus ossis incisive*, **mx** *os maxillare*, **snamx** *sutura nasomaxillaris*, **sfrmx** *sutura frontomaxillaris*, **splmx** *sutura palatomaxillaris*, **pzymx** *processus zygomaticus ossis maxillaris*, **szymx** *sutura zygomaticomaxillaris*, **cmx** *corpus maxillae*, **paom** *processus alveolaris ossis maxillaris*, **sepinta** *septa interalveolaria*, **ppom** *processus palatinus ossis maxillaris*, **svomx** *sutura vomeromaxillaris*, **pfom** *processus frontalis ossis maxillaris*, **fio** *foramen infraorbitale*, **ja** *juga alveolaria*, **na** *os nasale*, **sintna** *sutura internasalis*, **sfrna** *sutura frontonasalis*, **fr** *os frontale*, **pnaofr** *pars nasalis ossis frontalis*, **sfr** *squama frontalis*, **porofr** *pars orbitalis ossis frontalis*, **sintfr** *sutura interfrontalis*, **sfrlac** *sutura frontolacrimalis*, **sfrpa** *sutura frontoparietalis*, **lac** *os lacrimale*, **slacmx** *sutura lacrimomaxillaris*, **zy** *os zygomaticum*, **azy** *arcus zygomaticus*, **pzyotem** *processus zygomaticus ossis temporalis*, stemzy *sutura temporozygomatica*, **pr** *os parietale*, **ppr** *planum parietale*, **pnuch** *planum nuchale*, **ptem** *planum temporale*, ssag *sutura sagittalis*, **spripr** *sutura parietointerparietalis*, **socpr** *sutura occipitoparietalis*, **ipr** *os interparietale*, **sipr** *sutura interparietale*, **psp** *os presphenoidale*, **bsp** *os basisphenoidale*, **asp** *os alisphenoidale*, **osp** *os orbitosphenoid*, **syintsp** *synchondrosis intersphenoidalis*, **copsp** *corpus ossis presphenoidale*, **aopsp** *alae ossis presphenoidale*, **svosp** *sutura vomerosphenoidalis*, **ssppl** *sutura sphenopalatina*, **ssppr** *sutura sphenoparietalis*, **pocobsp** *processus occipitalis ossis basisphenoidalis*, **sspsq** *sutura sphenosquamosa*, **vo** vomer, **avo** alae vomeris, **svopl** sutura vomeropalatina, **pl** *os palatinum*, **lhopl** *lamina horizontalis ossis palatinum*, **sinacu** *spina nasalis caudalis*, **splmx** *sutura palatomaxillaris*, **antm** annulus tympanicus, **mal** malleus, **ro** os rostrale, **sq** os squamosum, **ssq** *sutura squamosa*, **cma** corpus mandibulae, **rma** ramus mandibulae, **fome** foramen mentale, **anma** *angulus mandibulae*, **pcor** processus coronoideus, **pcon** processus condylaris, **inuma** incisura mandibulae, **pe** os petrosum, **soc** *os supraoccipitale*, **boc** *os basioccipitale*, **exoc** *os exoccipitale*, **foma** foramen magnum, **syspoc** synchondrosis sphenooccipitalis, **conoc** condyli occipitales, **canhyp** canalis n. hypoglossi, **crnuch** *crista nuchae*, **pintpr** processus interparietalis, **socintpr** *sutura occipitointerparietalis*, **saorsp** *sutura aliorbitosphenoidalis*, **fo** *foramen ovale*, **ssqmas** *sutura squamosomastoidea*, **mattem** *matus temporalis*, **sseo** sutura supraexoccipitale, **ccon** *canalis condyliaris*, **fopr** *foramen parietale*.

**Figure 2:**
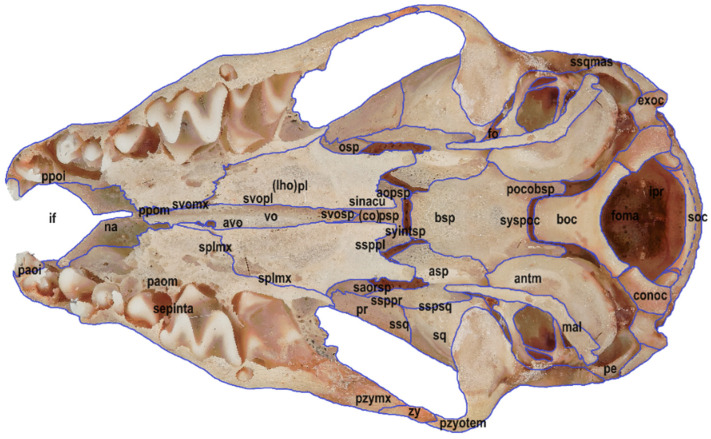
Ventral view of the skull in a juvenile specimen of *Myotis myotis*. The bones, sutures, and fusions are marked with lines. Abbreviations are explained below Figure 1.

**Figure 3:**
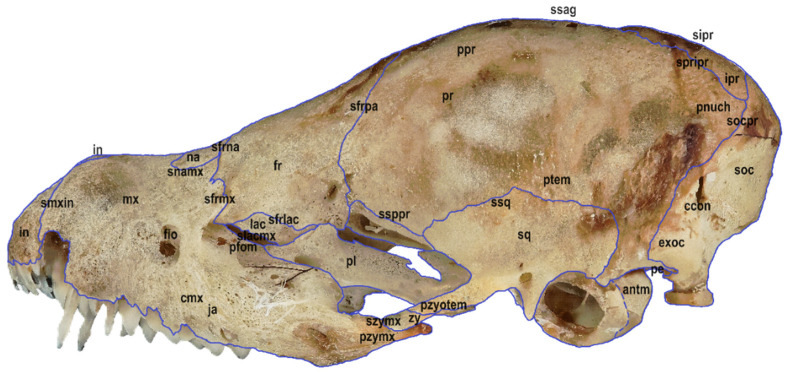
Lateral view of the skull in a juvenile specimen of *Myotis myotis*. The bones, sutures, and fusions are marked with lines. Abbreviations are explained below Figure 1.

**Figure 4:**
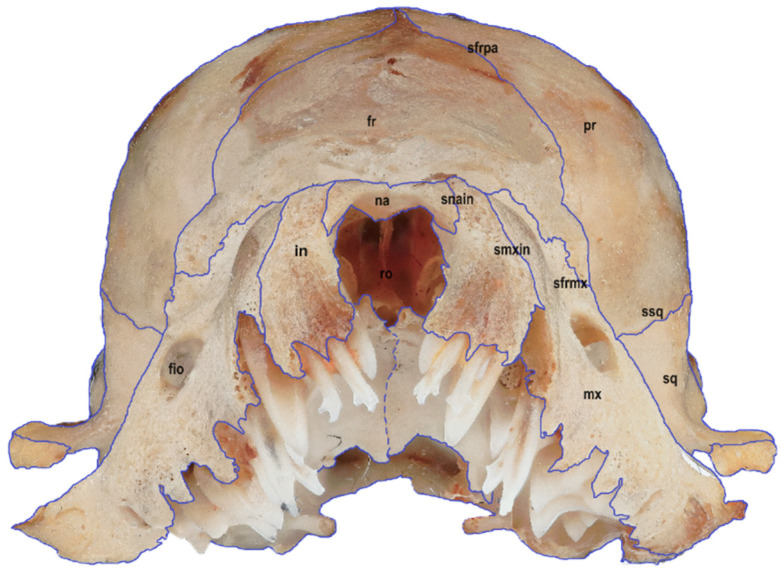
Rostral view of the skull in a juvenile specimen of *Myotis myotis*. The bones, sutures, and fusions are marked with lines. Abbreviations are explained below Figure 1.

**Figure 5:**
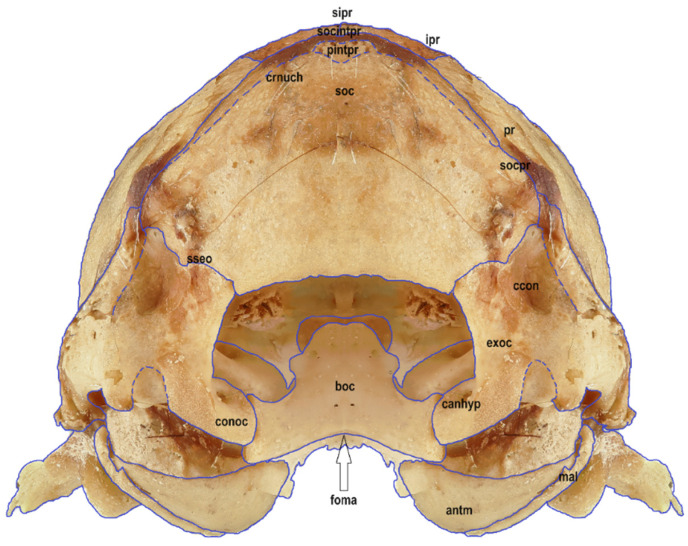
Occipital view of the skull in a juvenile specimen of *Myotis myotis*. The bones, sutures, and fusions are marked with lines. Abbreviations are explained below Figure 1.

**Figure 6:**
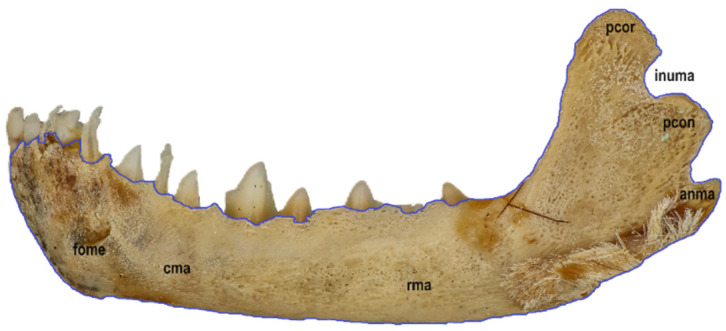
External view of the mandible in a juvenile specimen of *Myotis myotis*. The bones, sutures, and fusions are marked with lines. Abbreviations are explained below Figure 1.

Six macrophotographs of the skull of a juvenile *Myotis myotis* specimen were obtained as part of this study. The photographs allowed the authors to describe different structures of the skull that fade over several months as the bat develops. In total, 28 bones, 30 sutures, 13 processes and 5 foramens in the skull were described. The presence of the tear bone, *os lacrimale*, was confirmed. Below are six macro photographs of the skull of the selected individual: dorsal (Figure 1), ventral (Figure 2), lateral (Figure 3), anterior (Figure 4), posterior (Figure 5) and the mandible (Figure 6). The elements shown in the photographs are described in the next section of the article.

### 3.1. *Premaxilla* (os icisivum)

The premaxilla is the anterior-most bony element of the viscerocranium that forms its apex. The incisor bone is one of the smallest paired bones within the skull, and its main parts (left and right) are separated from each other. They do not fuse together at any stage of individual development because they do not share a common edge due to the rather large distance between them. Each part is located between the nasal bone (*os nasale*), to which it connects through the nasoincisal suture (*sutura nasoincisiva*), and the maxillary bone, to which it connects through the maxilloincisal suture (*sutura maxilloincisiva*) (Figure 1). The nasal bone, located along the midline of the skull, pushes both parts of the premaxilla outward. The nasal bone and the premaxilla together comprise the incisive fissure in the shape of an open rhombus or the letter U. As a result, the first pair of the upper incisors (*incisivi*)—I^1^ are free and do not touch each other (Figure 1). This gap in the central section of the premaxilla is considered a type of diastema. The premaxilla consists of the following elements:

- The body (*corpus ossis incisivi*), which borders the incisal gap from the front and serves as a free *processus alveolaris ossis incisive* that contains the alveoli of the two incisors of the upper dental arch (I^1^, I^2^). The upper alveolar process contains the space for two deciduous incisors and two permanent incisors (Figure 2);
- The *processus nasalis ossis incisivi*, which is the narrowest section that extends posteriorly and runs parallel to ¾ of the length of the longer edge of the nasal bone (Figure 1);
- The *processus palatinus ossis incisivi*, which forms part of the structure of the nasal portion of the bony palate (Figure 2).

### 3.2. *Jaw* (os maxillare)

The jaw is a paired bone, the largest within the viscerocranium. Due to pneumatization, it has a low ratio of mass and size. Three bones are located between the left and right parts of the jaw: the premaxilla (*os incisivum*), nasal bone (*os nasale*) and frontal bone (*os frontale*). These are connected to the jaw by the corresponding sutures: maxillary incisor (*sutura maxilloincisiva*), nasal-maxillary (*sutura nasomaxillaris*), and (very short) frontal-maxillary (*sutura frontomaxillaris*) (Figure 1). In addition, on the ventral side, the jawbone touches the palatal bone (*os palatinum*) through the palatomaxillary suture (*sutura palatomaxillaris*) (Figure 2). Laterally, the jaw transitions into the *processus zygomaticus ossis maxillaris* (Figure 1 and Figure 3), forming the anterior, nasal portion of the zygomatic arch. The jaw meets the zygomatic bone through the zygomaticomaxillary suture (*sutura zygomaticomaxillaris*). The jaw comprises a large body (*corpus maxillae*) and smaller processes as follows:

- The *processus alveolaris ossis maxillaris*, which contains the tooth sockets for the permanent teeth (I^2^, C^1^, P^3^, M^3^ × 2): two incisors (*incisivi*), one canine (*canini*), three premolars (*premolares*) and three molars (*molares*) belonging to the upper dental arch, the spaces for which are separated by the interdental septa (*septa interalveolaria*). The figure shows partial permanent teeth and deciduous teeth (i^2^, c^1^, p^2^ × 2) (Figure 2);
- The *processus palatinus ossis maxillaris*, which forms a large part of the bony palate. It is fused medially to the vomer via the vomeromaxillar suture (*sutura vomeromaxillaris*). The ventral surface of the palatal process shows a thin line, the incisive suture (*sutura incisive*) that is clearly visible in juvenile specimens. The incisive suture extends intranasally and laterally on both sides, up to the dental septum between the incisor and the canine (Figure 2);
- The semicircular *processus frontalis ossis maxillaris* (Figure 1);
- The triangular zygomatic process of the maxilla (*processus zygomaticus ossis maxillaris*), which is part of the zygomatic arch (Figure 1).

The outer, lateral surface of the maxilla contains the large, circular *foramen infraorbitale*, located near the third premolar (Figure 3). This foramen is located along the alveolar juga (*juga alveolaria*), which is a line of irregularities formed by the pressure of the dental roots against the outer wall of the jaw (Figure 3). In the general outline, the maxilla is a boat-shaped formation with a well-structured outer wall, which passes ventrally and centrally into the alveolar recess, which, in turn, gently descends into the inner wall, delineating the palate.

### 3.3. *Nasal Bone* (os nasale)

The nasal bone is a paired bone resembling open butterfly wings in shape. Its left and right parts are medially fused via *sutura internasalis* (Figure 1). This initially loose suture gradually hardens with age. As a result, the nasal bone forms a uniform plate that appears to be a single bone. The nasal bone can be described as an extension of the frontal bone, since together they form the “roof”, or the flat top of the nasal cavity. The front edges of the nasal bone border the back of the incisive fissure, whereas its lateral edges connect to the incisor bone through the nasoincisal suture (*sutura nasoincisiva*). Further on, the nasomaxillary suture (*sutura nasomaxillaris*) connects to the maxillary bone. The semicircular edge that encloses the nasal bone is the frontonasal suture (*sutura frontonasalis*) (Figure 1).

### 3.4. *Frontal Bone* (os frontale)

The frontal bone is paired and rectangular in shape. Its anterior part is involved in the formation of the nasal cavity and orbit, and its posterior part forms the vault of the cranial cavity. This allows the following areas to be distinguished within it (Figure 1):

– anterior = preorbital, constituting the *pars nasalis ossis frontalis*;
– middle = interorbital, serving as the *squama frontalis*;
– posterior = extraorbital, constituting the *pars orbitalis ossis frontalis*.

The left and right parts of the frontal bone are connected medially by the interorbital suture (*sutura interfrontalis*) (Figure 1), which solidifies at the early stages of development and is, therefore, not clearly visible. The anterior edge of the frontal bone is formed by two sutures: the frontonasal suture (*sutura frontonasalis*) and the frontomaxillary suture (*sutura frontomaxillaris*) (Figure 1). The lateral edge is formed by the frontolacrimal suture (*sutura frontolacrimalis*) (Figure 3), and the posterior edge is formed by the frontoparietal suture (*sutura frontoparietalis*) (Figure 1). The frontal bone is not visible when looking at the skull from below, because it is obscured by the palatal bone (*os palatinum*), the cuneiform bone (*os sphenoidale*), and the vomer.

### 3.5. *Tear Bone* (os lacrimale)

The tear bone is paired. It primarily encloses the eye socket from the nasolabial side of the skull. The tear bone is elongated and narrow, similar in shape to a rectangle. It is located between the frontal bone and the jaw. Unlike in other groups of animals, the *facies facialis* and *facies orbitalis* are not distinguished in bats of the genus *Myotis*, as the eye socket is too poorly defined. It connects to the neighboring bones by the lacrimomaxillary suture (*sutura lacrimomaxillaris*) (in juvenile specimens, there is a gap instead of a suture that becomes sutured over time) and the frontolacrimal suture (*sutura frontolacrimalis*) (Figure 3).

### 3.6. *Zygomatic Bone* (os zygomaticum)

The zygomatic bone is paired, and one of the smallest bones that comprise the bat’s skull. It is the central element of the zygomatic arch (*arcus zygomaticus*), which also makes it the connecting element between the viscerocranium and the cerebrum. From the external side (the space outside the skull) and internal side (the space enclosed by the zygomatic arch), the zygomatic bone does not touch the other structures of the skull. However, on the intranasal and caudal sides, the zygomatic bone is deformed by the following pointed processes, located opposite one another, that merge with it: the zygomatic process of the maxillary bone (*processus zygomaticus ossis maxillaris*) and the zygomatic process of the temporal bone (*processus zygomaticus ossis temporalis*). The zygomatic bone is attached to the maxillary bone through the zygomaticomaxillary suture (*sutura zygomaticomaxillaris*) and to the temporal bone through the lacrimomaxillary suture (*sutura temporozygomatica*) (Figure 1 and Figure 3).

### 3.7. *Parietal Bone* (os parietale)

The parietal bone is the largest paired bone of the skull that resembles a tuber in shape and accounts for a significant part of the cerebrocranial vault. Three *planes* can be distinguished: *planum parietale*, which is the back of the skull, *planum nuchale*, which extends to the posterior wall, and *planum temporale*, which forms part of the lateral wall. Medially, the left and right parts are connected by the sagittal suture (*sutura sagittalis*) (Figure 1). On the intranasal side, the parietal bone connects with the frontal bone through the frontoparietal suture (*sutura frontoparietalis*). Laterally, the anterior section of the parietal bone extends under the neurocranium to connect with the sphenoparietal suture (*sutura sphenoparietalis*) (Figure 2), which fuses the parietal bone with the alisphenoid. The lateral wall shows a connection to the squamosal through *sutura squamosa* (Figure 3). On the caudal side, it adjoins three bones as follows: angularly, the petrosal via *sutura petrosoparietalis* (Figure 3); posteriorly, the interparietal bone via *sutura parietointerparietalis*; and the occipital bone via *sutura occipitoparietalis* (Figure 5). Parietal openings may form on both sides of the parietal bone near the sagittal suture, but they are not always present. The parietal openings are clearly visible in adult specimens. A *foramen parietale* has been observed on each side (Figure 1), through which passes the parietal vein and, sometimes, also a branch of the occipital artery. Usually, these openings are symmetrical and form the mouth of the *matus temporalis*.

### 3.8. *Interparietal Bone* (os interparietale)

The interparietal bone is triangular and paired. Its base connects with the supraoccipital bone (*os supraoccipitale*) through *sutura occipitointerparietalis*, and its arms span the parietals. Laterally and intranasally along a 180° angle, both parts of the interparietal bone connect to both parts of the parietal bone through *sutura parietointerparietalis*. The interparietal bone forms the posterior, broad wedge of the cranial vault. Both parts of the interparietal bone are flat and plate-like in shape, without any depressions or protuberances, and are fused by *sutura interparietale* (Figure 1).

### 3.9. *Sphenoid* (os sphenoidale)

The sphenoid is an unpaired bone that consists of three main parts: the presphenoid (*os presphenoidale*), the basisphenoid (*os basisphenoidale*) and the alisphenoids (*os alisphenoidale*). Some alisphenoids function as the orbitosphenoid (*os orbitosphenoidale*). The sphenoid is located centrally in the base of the skull and touches almost all the other bones of the skull (except the nasal bone, lacrimal bone, and mandible). The sphenoid is responsible for cushioning; its structure helps distribute the strain forces arising within the skull evenly. The presphenoid fuses with the basisphenoid through the intersphenoidal cartilage (*synchondrosis intersphenoidalis*). The *corpus ossis presphenoidale* is square in shape, but diverges laterally into the irregularly shaped *alae ossis presphenoidale*. Each wing is an orbitosphenoid. The presphenoid connects to the vomer through *sutura vomerosphenoidalis* and to the palatine through *sutura sphenopalatina*. The sphenoparietal suture (*sutura sphenoparietalis*) emerges externally. In the caudal part of the juvenile skull, the presphenoid forms the *sphenoide fissure* at the touching point with the basisphenoid, which, in adult specimens, becomes fused, and laterally with the *alae ossis basisphenoidale*. The basisphenoid is approximately square in shape, with two *processus occipitalis ossis basisphenoidalis*. These processes show two *foramen ovale*, which are clearly visible in adult specimens. The basisphenoid forms the base of the cerebrum. The lateral edge of the alisphenoid forms a broad, rough surface that connects it through *sutura sphenosquamosa* to the squamosal. The orbitosphenoid is a small, roughly oval bone that connects with the alisphenoid through *sutura aliorbitosphenoidalis*. The basisphenoid is a large bone and one of the elements of the sphenoid complex. The basisphenoid consists of a body and a pair of wings, i.e., the alisphenoids, which are fused to the body of the basisphenoid (Figure 2). On the dorsal surface of the sphenoid is the *sella turcica*, the most clearly marked structure of which is the pituitary fossa. The pituitary fossa is delineated intranasally by the *tuberculum sellae*, and posteriorly by the *dorsum sellae*. These elements are not visible in the figures.

### 3.10. *Vomer* (vomer)

The vomer is a narrow, long, unpaired bone, shaped like the letter V. The vomer is located medially in the ventral part of the nasal capsule, and, consequently, it touches the nasal crest of the palatine with its lower edge, i.e., the crest of the vomer (crista vomeris). This vomer consists of two narrow plates running parallel to each other, from which the wings of the vomer (alae vomeris) diverge laterally. The left and right plates of the vomer connect to each other at an acute angle. The vomer connects to the palatal process of the maxilla through the vomeromaxillary suture (*sutura vomeromaxillaris*) and the palatine through the vomeropalatine suture (sutura vomeropalatina). Posteriorly, the vomer forms small pterygoid processes that adjoin the presphenoid. In total, the vomer forms two intranasal wings and two caudal wings (Figure 2).

### 3.11. *Palatine* (os palatinum)

The palatine is a paired bone that forms the bony palate and the posterior nostrils. It is located between the maxilla, the pterygoid, and the presphenoid and contains the horizontal lamina (*lamina horizontalis ossis palatinum*) and the perpendicular lamina (*lamina perpendicularis ossis palatinum*). The former occupies a larger area than the latter and is a flat bone that fills the space between the upper bilateral dental arches. It participates in the formation of the secondary bony palate and nostrils. At the end of the horizontal lamina is the caudal nasal spine (*spina nasalis caudalis*). It is located perpendicularly to the vertical lamella, which, in turn, is a caudal element attached to the horizontal lamella. The vertical lamella is part of the lateral walls of the nasal passage. The palatine connects to the presphenoid and the alisphenoid through the sphenopalatal suture (*sutura sphenopalatina*), to the maxilla through the palatomaxillary suture (*sutura palatomaxillaris*), and to the vomer through the vomeropalatine suture (*sutura vomeropalatina*) (Figure 2).

### 3.12. *Ectotympanic* (annulus tympanicus)

The ectotympanic is a spherical, paired bony formation located on the ventral side of the skull. Its medial part resembles a dorsally tapering ring and overlaps the basisphenoid (Figure 2 and Figure 3).

### 3.13. *Auditory Ossicles* (ossicula auditus)

The auditory ossicles are a set of small bones. They are also light due to numerous perforations, clearly visible in adult specimens. The auditory ossicles constitute the bony elements of the middle ear.

### 3.14. *Malleus* (malleus)

The malleus is a small bone compared to the skull as a whole, although it is the largest of the auditory ossicles of the middle ear. It connects to the anvil through the head of the malleus (caput mallei). On the opposite side is the rostral process (processus rostralis) (Figure 2), which adjoins the tympanic membrane. On the inner surface of the body of the malleus is a large depression. The name of this bony element is related to its function; the malleus transmits the vibrations of the sound waves from the tympanic membrane to the incus.

### 3.15. *Incus* (incus)

The incus is a medium-sized auditory ossicle of the middle ear. The incus has an articular surface that connects it with the malleus on one side, and, on the other, a process that connects it with the stapes. It also has a small, shallow groove on the side of the rostrum.

### 3.16. *Stapes* (stapes)

The stapes is the smallest auditory ossicle. It consists of the head (*caput stapedis*), base (*basis stapedis*), *crus anterius stapedis* and *crus posterius stapedis*. The stapes transmits vibrations from the tympanic membrane (*membrana tympani*) to the inner ear. 

### 3.17. *Rostral Bone* (os rostrale)

The rostral bone is located in the anterior part of the skull. It is a spatial bone resembling a pyramid (Figure 4). The rostral bone is a visceral bone, which is a type of bone associated with specific organs. It supports the nasal cavity and provides resilience to the rostral part of the skull. 

### 3.18. *Squamosal* (os squamosum)/Temporal Bone (os temporale)

The squamosal is a relatively large, paired bone that forms the lateral wall of the cerebrum. It is a flattened, squamous structure with a zygomatic process diverging from it. It consists of three parts: the pars squamosa, pars petrosa, and pars tympanica. The outer surface of the temporal is convex and smooth. On the inside are depressions created by the deformation of the posterolateral part of the cerebral hemispheres and the vestibular system. The petrous and tympanic parts of the squamosal enclose the structures of the middle and inner ear. The zygomatic process of the temporal bone is part of the zygomatic arch, as described in the section concerning the jugal. The processes that comprise the arch are connected to one another through the small jugal. In young specimens, the temporal fossa is visible on the surface of the squamosal. The temporal fossa contains holes that fuse together into a continuous bone over time. The squamosal connects to the parietal bone through sutura squamosa (Figure 3), the sphenoid through sutura sphenosquamosa, and the petrosa through sutura squamosomastoidea (Figure 2). On the ventral side of the epiphysis of the zygomatic process is the mandibular fossa, which provides connection with the articular process of the mandible. From the outside, the mandibular fossa is delineated by the articular tubercle. A temporal canal runs through the temporal bone that ends with a large opening in the temporal canal and/or several small openings on the side of the articular tubercle (Figure 3).

### 3.19. *Mandible* (mandibula)

The mandible is a paired bone. Its left and right parts are connected by the intermandibular synchondrosis (synchondrosis intermandibularis), which, in contrast to a suture, is not visible. The mandible is composed primarily of a robust body (corpus mandibulae) and the ramus of the mandible (ramus mandibulae) that faces the zygomatic arch. It contains the alveoli for the teeth of the lower dental arch (I_3_, C_1_, P_3_, M_3_ × 2): three incisors (incisivi), one canine (canini), three premolars (premolares) and three molars (molares). And for deciduous teeth (i_3_, c_1_, p_2_ × 2), the following surfaces can be distinguished within the mandible: facies alveolaris, facies buccalis, facies lingualis and facies labialis. The lingual and labial surfaces meet at the alveolar margin (margo alveolaris) of the incisors and canines. Some buccal teeth (the molars and premolars) are located within the lingual and buccal surfaces. A single mental foramen (foramen mentale) is clearly visible. On the caudal–ventral side, the mandible forms a semicircular angle of the mandibular (angulus mandibulae), which is poorly defined in juvenile specimens. Two processes develop over the angle of the mandibular: the coronoid process (processus coronoideus) and the mandibular condyle (processus condylaris). These are separated by the incisura mandibulae. On the inner and outer surfaces of the ramus of the mandible are numerous shallow depressions, which are the vestiges of the masseter muscle attachment (Figure 6).

### 3.20. *Petrosal* (os petrosum)

The petrosal is a well-developed, dome-shaped bone located on the ventral side of the skull (Figure 2). The petrosal contributes to protecting the sensitive structures of the organ of hearing and balance. It protects the inner ear and, consequently, the entire vestibular system.

### 3.21. *Occipital* (os occipitale)

The occipital is an unpaired bone composed of three parts, the supraoccipital (*os supraoccipitale*), the basioccipital (*os basioccipitale*), and the extraoccipital (*os exoccipitale*), which is separated into left and right parts. The occipital is a component of two walls of the skull, specifically, the caudal, terminal section of the base of the skull and the posterior wall. On the ventral side of the skull, at the base of the occipital, is the foramen magnum, literally, “the great hole”. The name is justified, as it is the largest opening of the skull. It emerges at the junction of all occipital bones. It has a regular ellipsoidal shape and marks the center of the occipital. The part located above the foramen magnum is the supraoccipital, also referred to as *squama occipitalis*. In turn, below the foramen magnum, i.e., on the lower wall of the skull, is the basioccipital, or *pars basillaris*. The supraoccipital and basioccipital are flat. The basioccipital is divided into three parts, namely the body and two lateral processes facing the dorsolateral part of the skull. The width of the basioccipital is directly proportional to the degree of development of the vestibular system. On the dorsal surface of the body of the basioccipital is a poorly defined longitudinal groove that houses the medulla oblongata. The basioccipital connects to the basisphenoid through *synchondrosis sphenooccipitalis*. The dorsal surface of the body of the basaloccipital bone, i.e., the surface facing the brain cavity, is slightly concave, whereas its ventral side is slightly convex. Both surfaces are smooth. The supraoccipital has a convex outer surface and a concave inner surface. It is the largest bone of the posterior part of the skull. Its lower edge adjoins *foramen magnum*. The inner surface is deformed by the clearly preserved imprint of the cerebellar hemispheres. It connects laterally to the left and right parts of the extraoccipital through *sutura supraexoccipitale*, the parietal bone through *sutura occipitoparietalis*, and the interparietal bone through *sutura occipitointerparietalis*. Between the upper and lower occipital bones is the slightly semicircular extraoccipital (*os exoccipitale*), which can be considered a paired bone due to the *foramen magnum* separating it into *partes laterales*, or the left and right bones. A bony protrusion is present on both parts, which extends along the left and right edges of the *foramen magnum*, respectively. The protrusions are symmetrically relative to each other and are called the occipital condyles (*condyli occipitales*). A small opening is visible in each of them, which is the entrance to *canalis n. hypoglossi*. A cervical process emerges outwards from each occipital condyle. Between the occipital condyles is the jugular notch. The nuchal crest (*crista nuchae*) emerges from the upper edge of the supraoccipital to the extraoccipital. The nuchal crest resembles the arms of a triangle, but appears gradually with age, once the ossification of this part of the skull has progressed. It is not visible in juvenile specimens, and the entire outer surface of the occipital is smooth. At the junction between the supraoccipital bone and the interparietal bones, a triangular *processus interparietalis* is present in the posterior part of the cranial vault. This is another clearly visible element, present mainly in older individuals. The occipital creates *sutura occipitoparietalis* and *sutura occipitointerparietalis* at the junction with the adjacent bones. The extraoccipital has two relatively independent parts, the *pars occipitalis* and the *pars temporalis*, the boundary of which runs dorsolaterally along the occipital condyles, cervical processes, and jugular notch. The lateral inner edge of the extraoccipital delineates the *foramen magnum*. The *canalis condyliaris* runs bilaterally through the extraoccipital (Figure 5).

### 3.22. *Pterygoid* (os pterygoideum)

The pterygoid is a small, paired bone. It consists of a horizontal plate adjoining the body of the presphenoid and a vertical pterygoid process that forms the lateral walls of the posterior nostrils. The *hamulus pterygoideus* is the free end of the pterygoid, which, in *Myotis myotis,* is poorly defined.

### 3.23. *Ethmoid* (os ethmoidale)

The ethmoid is a small, unpaired bone that is not visible from the outside and divides the brain cavity from the nostril cavity of the skull (Figure 4). The lamina of the ethmoid is punctured by numerous holes. The dorsal edge slants slightly anteriorly. A perpendicular plate facing the nasal cavity connects to the septum. The primary function of the septal turbinate is to increase the surface area of the nasal cavities in order to more effectively warm up and humidify the air passing from the nasal cavity to the lungs.

## 4. Discussion

The skull is a unique part of the axial skeleton of mammals. It is composed of a set of cartilaginous and solid bones with different origins and functions. This set can be considered a macro-functional unit that protects the internal structures (e.g., the brain, the organs of sight, hearing, smell, taste, and balance, etc.), as well as various elements of the respiratory, circulatory, nervous, and digestive systems. It is also the site of the attachment of many muscles, both on the inside and on the outside. An integral part of the skull is the powerful dental apparatus located in the mandible and the maxilla. The concentration of so many structures within the skull determines its morphological complexity and its overall structure, which, in turn, translates into a considerable variety in its individual elements between the representatives of *Myotis myotis* and other species of the order Chiroptera. Consequently, describing and analyzing the structural elements of the skull provides material for use in many disciplines of science, including systematics, statistics, and descriptive analysis.

There are few studies on the bone structures of the bat skull, especially in juvenile specimens of *Myotis myotis*. Frick [24] conducted a histological assessment of embryos, which, to this day, remains the most detailed paper of its kind. 

The bone structures of juvenile specimens are unossified but, nonetheless, well-developed, and become fully fused 2–3 months after birth. The ossification finishes immediately before the bats acquire the ability to fly [16]. This is likely due to the key changes that take place in the morphology and pressure of the skull in order to quickly improve occlusal performance during ontogenesis [25].

Analysis of the skeletal structures in juvenile specimens helps to identify many features characteristic of *Myotis myotis* that are not discernible on the skull of adult specimens. The skull shows a tendency for the orbit to move in the nasal direction. Furthermore, the jaw is reduced, the ectotympanic is a continuous element, and the entire eardrum is a free structure that does not connect with the skull. According to Freeman [16], the bat skull consists of 24–28 bones (17–19 paired and 6–7 unpaired). Almost all of the bones are firmly fused together through sutures or synchondroses (with the exception of the hyoid apparatus and the ossicles, which are freely located in the middle ear). The bony elements of the skull are markedly reduced compared to other amniotes [26]. The skull of a contemporary adult mammal contains about 30 free elements [15,27].

The temporomandibular joint, created by the squamosal and the mandible, is the only structure that retains mobility between the elements of the skull. As found by Freeman [16], vestigial frontal fontanel was also found in the present study. The reduced number of skull bones in adult specimens results from the fact that some of them combine into complexes. For example, the lacrimal bone is connected with the maxilla [16]. The interparietal bone is monolithic (as with the Megachiroptera) [2]. Another difference in skull structure between juvenile and adult specimens is an underdeveloped sagittal crest. This study concerned an intermediate stage of the skull with well-developed bony structures. Some bones of the skull in juvenile specimens are underdeveloped, and some are reduced. Currently, there are not explanations for the removal of interosseous sutures; however, current research provides some insights that may clarify this issue. In almost all the species of the order Chiroptera, the interosseous sutures fuse before active flight begins. The premaxillary and nasal bones do not fuse with the monolithic dome of the skull only in some representatives of the order [15]. The research allowed for the general hypothesis that bats evolved from their ancestors together with Insectivora, which is confirmed by genetic studies [28,29,30]. Thus, the structural characteristics of the cartilaginous elements of the skull of Insectivora, Chiroptera, and Primates show a certain similarity between them. The Insectivora and Microchiroptera display a stronger similarity than the Macrochiroptera and Primates [31]. This leads the authors of this study to assume that the representatives of these orders evolved from one or more common ancestors [30].

The most notable aspect of the final structure of the skull in the Chiroptera and Primates is the increased volume of the braincase, which was previously observed in the cartilaginous skull in both orders and confirmed by previous studies [32,33,34]. Also notable is the general shortening of the viscerocranium in many bats, especially the Microchiroptera [16], which is the most prominent in vampires (Desmodontidae). The length of the viscerocranium (relative to the brain) is minimal, which is, to an extent, reflected in the maximum number of permanent teeth in bats, i.e., 38 [35]. Overall, Chiroptera and Primates have a tooth type similar to that of their ancestors (similar to Insectivora). It should be noted that the skull of *Myotis myotis* and most Microchiroptera and Primates show a structural similarity in terms of atavistic and specialized features. The atavistic features include a similar general cartilaginous structure of the skull, especially the nasal cavity and braincase, primitive teeth, the alisphenoid and orbitosphenoid fused with the presphenoid and basisphenoid, respectively, a similar structure of the elements of the auditory apparatus, and a similar structure of the incisors. In turn, the specialized features include a shortened viscerocranium, increased volume of the braincase, alisphenoid being larger than the orbitosphenoid, a tendency for the orbit to move towards the front of the skull, and the effacing of the interosseous sutures.

Only a few publications address all the stages of the embryogenesis of the skull [24], including in *Nyctalus noctula* [16,36]. Partial embryogenesis of the skull (one or several stages) was investigated in nine bat species [16]. For example, the interparietal bone is unpaired in the analyzed representatives of Megachiroptera, but paired in a vast majority of Microchiroptera.

Below is a comparison of the most important body elements assessed in this study with previous studies:

(1) The frontal bone (*os frontale*) is not connected with the zygomatic arch. These two bones are connected to each other in Primastes, Artiodactyla, and some Carnivora and Hyracoidea, creating a continuous orbital annulus [37,38,39];
(2) One of the most specific features of Microchiroptera is the orbitotemporal area. The temporal fossa (*fossa temporalis*) is characteristic not only of Chiroptera, but also lower Placentalia, primarily Insectivora. The border between the orbit and the temporal fossa is poorly defined in Carnivora, Lagomorpha, Rodentia, Pinnipedia, and Tubulidentata, but clearly defined in Primates and Artiodactyla [37,38,39];
(3) In most mammals, the zygomatic arch (*arcus zygomaticus*) is created by the temporal process of the jugal and the zygomatic process of the temporal bone. However, in the assessed species, the zygomatic arch is created by the zygomatic processes of the maxillary and the squamosal, which are connected through a small jugal. Most Insectivora have a similar structure [40];
(4) The nasal bones (*os nasale*) are clearly separated from one another in juvenile specimens but fused together in adult animals. In many Megachiroptera, the nasal bones remain free throughout their entire life, but, in some species, they form a single, unpaired nasal bone [3,41,42,43,44]. The nasal bones are usually narrow and elongated in Megachiroptera, and wide and short in Microchiroptera [16,45]. The authors of this study do not agree with the belief that the nasal bones are short. According to Starc [46], bats tend to reduce the nasal bone. Its advanced structure indicates a well-developed sense of smell;
(5) Lacrimal bone (*os lacrimale*)—subject literature provides two explanations for its absence in some bats; the lacrimal bone was reduced, even though the nasolacrimal canal has been retained [47], or it has fused with the coronal process of the maxilla [24]. In most of the analyzed bats, the lacrimal bone is located in the anterior part of the orbit, which, according to H. Frick [24], is absent in *Desmodus*, *Myotis*, and *Rhinolophus*. The authors of this study believe that the lacrimal bone is present in the representatives of *Myotis myotis*;
(6) The alisphenoid (*os alispenoidale*) is much larger in most bats than the orbitosphenoid [16];
(7) The tympanic bone (*os tympanicum*) is horseshoe-shaped, relatively large and never fuses with the rest of the cranial bones (except for the malleus, with which it fuses at later stages of postnatal ontogenesis) [16]. A similar structure to the tympanic bone is observed only in Insectivora. Not all analyzed animals showed a fusion of the tympanic bone with the skull [48]. The hearing organ of bats contains a large endolymphatic sac. The structure of some elements of the skull is related to a bat’s echolocation ability [6,49];
(8) The occipital condyles in newly born bats are usually underdeveloped and resemble small lumps. Their degree of development varies between all terrestrial Placentalia;
(9) The structure of the premaxilla (*os premaksilare*) varies considerably among the Chiroptera order, and its presence has not been explained yet;
(10) In most mammals, the interparietal bone (*os interparietale*) fuses with the supraoccipital bone. It is present in some Insectivora and Rodentia in the form of an unpaired bony plate [47,50,51]. However, in those animals, the interparietal bone is much smaller than in many bats. The interparietal bone is unpaired in bats such as the Pteropidae, Rhinolophidae, and Hipposideroidae [13,16,43,44], and even in other bats, including *Myotis myotis*;
(11) The occipital region consists of four independent bones [16], specifically the basioccipital, the supraoccipital and two parts (left and right) of the exoccipital. The basioccipital bone (*os basioccipitale*) in Microchiroptera forms a large plate;
(12) The crown of deciduous teeth is single-, double- or triple-lobed, which is considerably different from the crown of permanent teeth. Exceptions include the representatives of the families Rhinolophidae and Hipposideroidae, in which deciduous teeth do not erupt and are resorbed as early as in the prenatal period of ontogenesis [41,42,43,44,45,46,47,48,49,50,51,52,53,54,55,56,57,58,59,60,61,62,63,64,65,66];
(13) The secondary palate is a structure typical, according to Kovtun and Likhotop [15], among the structures that took part in the early stages of the formation of the palate in the division of the oral cavity into the nasal and secondary oral cavities, the pterygoid sides, provomerum and vomer are also called. Later studies identified the premaxilla as the inner lamina of the palatal process of the premaxillary bone [67,68]. According to Kovtun and Likhotop [69], the premaxillary bone does not contain a palatal process and does not participate in the formation of the bony palate. Consequently, among the analyzed bats, only the Vespertilionidae are characterized by the participation of the vomer in the formation of the bony palate. To summarize the above findings, while it seems possible to distinguish the five structural types of the bony palate among mammals, in the Vespertilionidae, the formation of the bony palate involves the palatal bone, the maxilla, and the vomer;
(14) Incisive fissure (*incisive fissure*)—in Megachiroptera, it resembles a large palatal opening due to the fusion of the premaxillary bones. In *Myotis myotis*, because the premaxillary bones do not fuse, the incisive fissure is located in the central and rostral part of the skull, rather than on the palatal surface. Consequently, it can be described as a diastema typical of this species.

Despite the large number studies on the skull, some aspects of the morphology of bats remain unexplored. However, the authors of this paper believe that there are many more aspects that still require research and analysis.

For example, Koyabu [27] highlights that patterns of ossification, cranial sutures, and taxon-specific neomorphic bones of the skull have almost never been investigated, and that further research would provide insight on the structure of mammalian skulls. The authors of this paper share Koyabu’s [27] opinion that the skull may contain bones that have not yet been confirmed or discovered by science, which suggests a need for further research, especially concerning the skulls of bats. This results from the high interspecies variation in the heterochrony of ossification in mammals [69,70,71]. The causes of the overall simplification of the skull and the reduction in the number of its elements are, in many cases, still unclear. The bones of the skull ossify at different rates among mammalian species [15]. As has already been mentioned, the skull ossifies extraordinarily rapidly in bats compared to other mammals, which needs explaining.

Another aspect worth pursuing is the high variety in the fusion of cranial sutures. Sutures are defined anatomically as the fibrous joints between the elements of the skin, whereas synchondroses are defined as the cartilaginous joints between the cartilages of the bones of the skull [72]. Pedersen [73] described a general sequence of ossification of the skull in bats, including *Myotis myotis*. Furthermore, a growing amount of evidence suggests that the ossification sequence is highly species-specific and variable among species [28,70,72,74]. In contrast to many land mammals, the skull of most adult bats does not show clearly defined sutures. On the other hand, the juvenile skull analyzed in this study does show the sutures.

Because the authors did not find the names of the sutures in the literature, they proposed their own terminology for these structures. To date, comparative anatomy has defined thousands of terms describing anatomical parts of vertebrates. However, handbooks often do not clearly define all the elements of the skull [27]. Consequently, it is worth demonstrating the perspectives for typically ignored problems related to the homology, development, and conservatism of the mammalian skull in order to encourage research in these areas. Research on the basic structure of the mammalian skull is generally assumed to be complete [26]. However, the case analyzed in this study suggests otherwise, pointing out the need for more detailed research. The authors of this study used the terms squamosal (*os squamosum*) and temporal bone (*os temporale*) interchangeably to describe the lateral wall of the skull. The findings presented in this study are of fundamental importance to the understanding of the structure of the skeletal system and the relationships between its elements. This will provide insight on the distribution of each structure in both juvenile and adult specimens to be investigated.

## 5. Conclusions

There are few studies analyzing the skull of bats, especially in the juvenile specimens of *Myotis myotis*. Analysis of the bony elements of the juvenile specimen helped to identify a number of features characteristic of *Myotis myotis* that are not visible in adult specimens. The reduced number of skull bones in adult bats is related to their fusion into complexes. According to different researchers, the skull of the bat consists of 24–26 bones (17–19 paired and 6–7 unpaired). Almost all the bones are strongly connected to one another through sutures or synchondroses.

This study presented a detailed morphological and anatomical description of the skull of a young specimen of *Myotis myotis* (Borkhausen, 1797). The authors used the collection of the Upper Silesian Museum in Bytom (Poland). Juvenile skulls are difficult to preserve and often incomplete. Previously inconsistent terminology of bones, sutures, and other cranial structures was unified, which provided insight on the distribution of each structure in both juvenile and adult specimens. The description and analysis filled in the gaps in knowledge about the cranial structures of *Myotis myotis* and the representatives of the family Vespertilionidae.

## Data Availability

The original contributions presented in the study are included in the article.
